# Global horizontal irradiance data for 12 stations in Mauritius, Rodrigues and Agaléga

**DOI:** 10.1016/j.dib.2024.110985

**Published:** 2024-09-30

**Authors:** Yogesh Beeharry, Ravish Gokool, Yatindra Kumar Ramgolam, Aatish Chiniah

**Affiliations:** University of Mauritius, Reduit 80837, Mauritius

**Keywords:** Solar radiation, Solar energy data, Solar irradiance measurements, Renewable Energy, Pyranometer, Mauritius

## Abstract

This data article presents the Global Horizontal Irradiance data acquired at a temporal resolution of one-hour intervals from 12 sites around the island of Mauritius, Rodrigues and Agaléga. These data were collected over different durations for different sites. The data acquisition process was conducted via Base stations consisting of a TMF100 Data logger, an MS-802 EKO Pyranometer, a Silicon Irradiance Sensor with an integrated temperature sensor, and an Air Temperature Sensor for data collection and a GPRS (General Packet Radio Service) modem for transmission of collected data to an FTP server. The retrieved data from the FTP (File Transfer Protocol) server had to undergo time alignment as a misalignment of 8 h was observed.

Such dataset can be used for solar energy resource analysis and forecasting, photovoltaic system performance analysis as well as bringing awareness regarding the installation of commercial solar PV projects. This data set will also help to facilitate data-driven decision-making processes and enhance the understanding and utilization of solar energy resources.

Specifications Table

**Table:** 

Subject	Energy: Renewable Energy, Sustainability and the Environment.
Specific subject area	Forecasting Global Horizontal Irradiance for efficient utilization of solar energy resources
Type of data	.csv. Raw data and Analyzed data
Data collection	The data acquisition was conducted by base stations located at nine sites across the island of Mauritius, 1 site in Agaléga Island and 2 sites in Rodrigues Island. The base station consisted of a TMF100 Data logger, an MS-802 EKO Pyranometer, a Silicon Irradiance Sensor with an integrated temperature sensor, and an Air Temperature Sensor. The TMF100 Data Logger is connected to the MS802EKO Pyranometer which is responsible for Solar Irradiance measurements. The logging of the data was set to 1-h intervals. IP68 black connectors were used for signal transmission and GPRS modem was used for transmission of data to the FTP server. The data was then downloaded from the FTP server and cleaned to remove any erroneous characters. The initial data, in .txt format and delimited with commas and hash symbols, was processed using a Python script. This script read the data, filtered out unnecessary information, and retained only the Global Horizontal Irradiance (GHI) values. Headers for Timestamps, Site Name, Latitude, Longitude, and Global Horizontal Irradiance were added to the data. An eight-hour time alignment offset was applied, and nighttime data (before 7AM and after 7PM) was removed. Finally, the cleaned and processed data was saved in .CSV format, with filenames indicating the site's island, name, latitude, longitude, starting date, and ending date.
Data source location	Agalega (10.387° S, 56.618° E) Bramsthan (20.214° S, 57.737° E) Curepipe (20.320° S, 57.526° E) Ferney (20.368° S, 57.698° E) Goodlands (20.040° S, 57.655° E) Grenade (19.685° S, 63.480° E) LaMivoie (20.345° S, 57.364° E) Mahebourg (20.408° S, 57.697° E) Nicolay (20.152° S, 57.513° E) Port Mathurin (19.682° S, 63.419° E) Rose-Hill (20.242° S, 57.476° E) Souillac (20.501° S, 57.523° E).
Data accessibility	Repository name: Science Data Bank Data identification number: https://doi.org/10.57760/sciencedb.08095 Direct URL to data: https://www.scidb.cn/en/detail?dataSetId=2b499b91a4464fffa9f60fc8b51da03e&version=V2
Related research article	none*.*

## 1 Value of the Data


 

• Solar irradiance data from various locations in Mauritius offer valuable insights into regional solar energy availability, aiding renewable energy project planning and optimization.
• These data allow researchers to analyse long-term solar irradiance trends and fluctuations, aiding studies in meteorology, climatology, renewable energy technology, and environmental science.
• The information can be utilized for future solar energy project monitoring or compared with model-predicted datasets, enhancing the accuracy and applicability of renewable energy planning efforts.

## 2 Background

The motivation behind compiling this dataset is to help electricity distribution network operators to optimize energy production. By providing comprehensive data, operators can strategically plan for the variability of solar irradiance. Knowledge of solar irradiance data at different locations around the islands (Mauritius, Rodrigues and Agaléga) enables electricity network operators to make necessary adjustments and seamlessly integrate other sources of electricity, in order to ensure an efficient energy grid. Additionally, this data supports the planning and installation of commercial photovoltaic projects by providing information on solar energy availability around these islands.

## 3 Data Description

Global Horizontal Irradiance (GHI) measured in Watts per square meter (W/m²) was collected from 12 base stations across the islands of Mauritius, Rodrigues and Agaléga at intervals of 1-hour. The time periods covered are: 2017–2023 on Mauritius island, 2017–2019 on Rodrigues island and 2017–2018 on Agaléga island. The sampling process typically involves deploying high-quality pyranometers installed in locations free from obstructions like buildings or trees that may cause shading or reflection. The sites from which data were collected are: Agaléga, Bramsthan, Curepipe, Ferney, Goodlands, Grenade, La Mivoie, Mahébourg, Nicolay, Port Mathurin, Rose Hill and Souillac.

The Global Horizontal Irradiance dataset [1] comprised of:

• Timestamps: Date (YYYY-MM-DD) and time (HH:MM), recorded in UTC+04:00.
• Site Name: Name of the data collection site.
• Latitude: Latitude in degrees.
• Longitude: Longitude in degrees.
• Global Horizontal Irradiance: Measured in W/m².

**Fig. 1:**
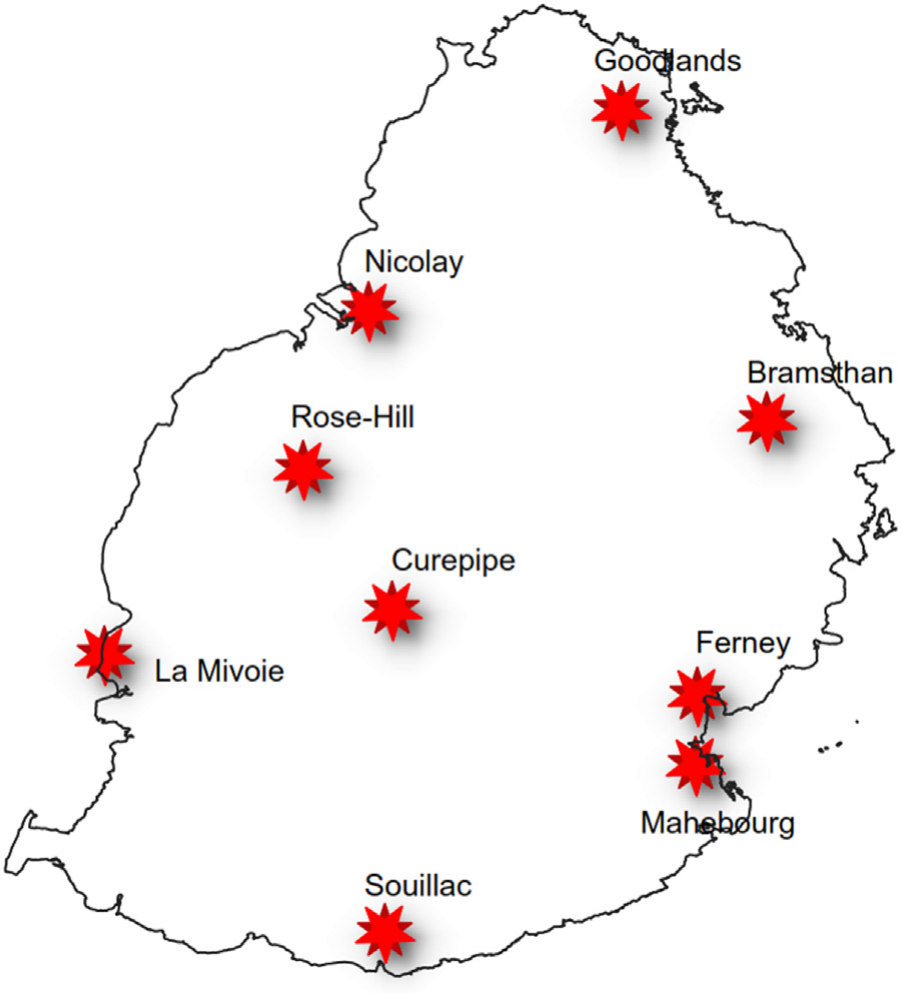
Site locations for Mauritius.

**Fig. 2:**
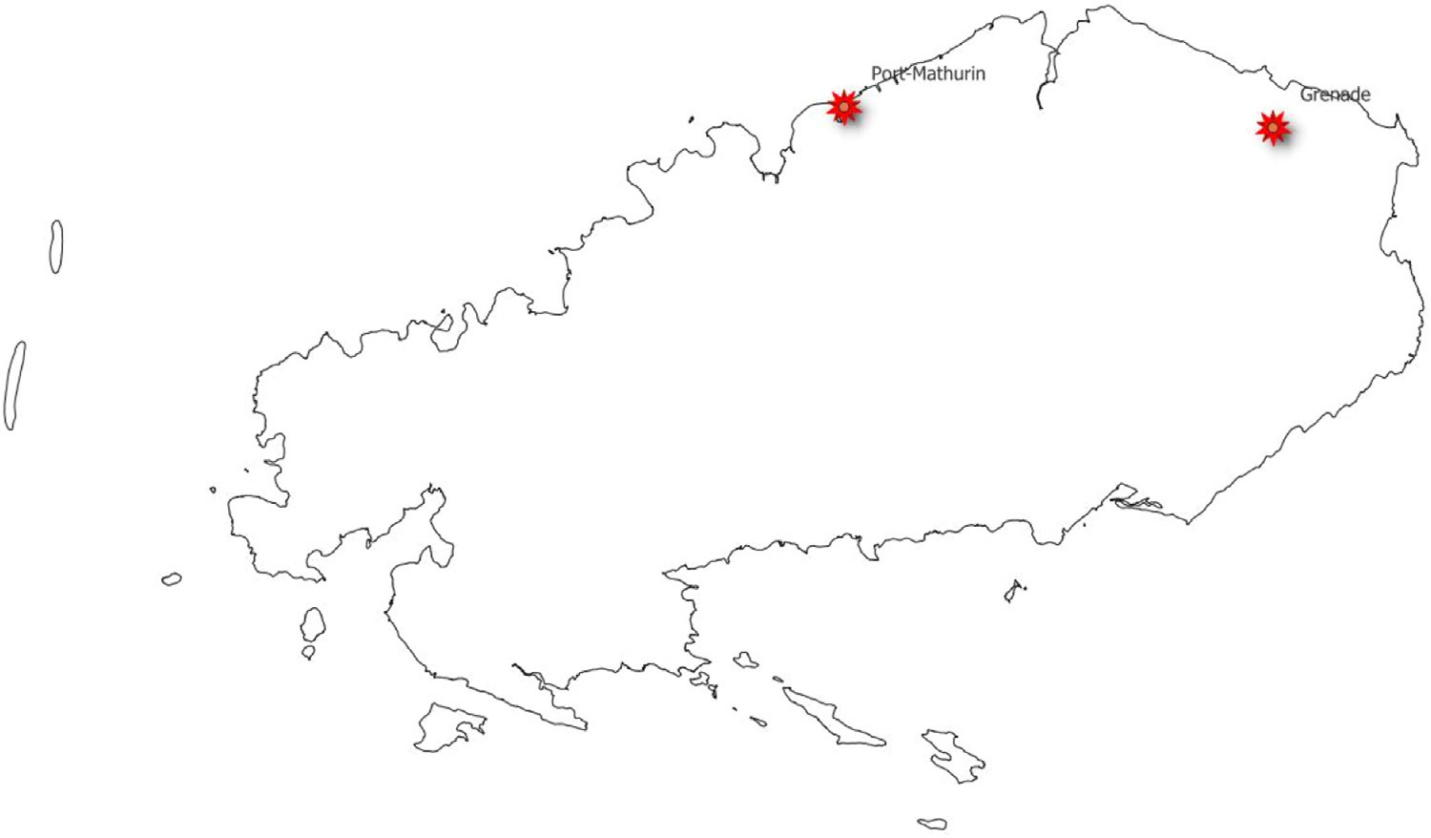
Site locations for Rodrigues.

**Fig. 3:**
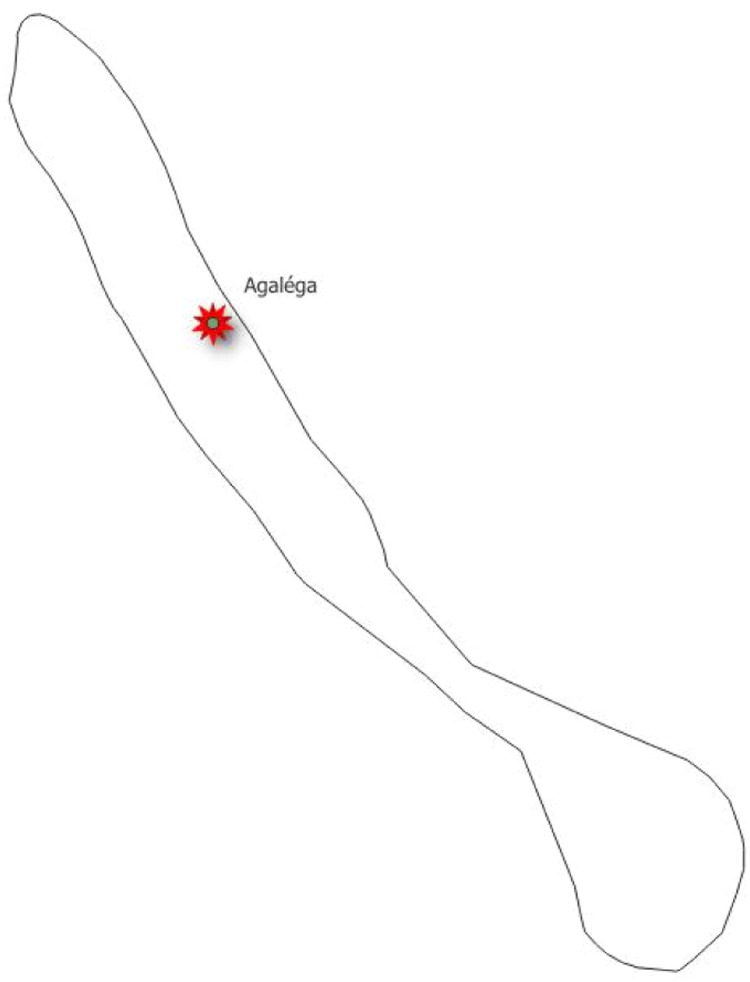
Site locations for Agaléga.

A sample data point is shown in Table 1. The GIS-based maps for Mauritius, Rodrigues and Agaléga are shown in Fig. 1, Fig. 2, Fig. 3 respectively. The locations at which the data logging systems have been deployed are also shown for each one.

**Table 1:** Sample data point for site Bramsthan.

Timestamp	Site Name	Latitude	Longitude	Global Horizontal Irradiance
2020–11–30 12:00	Bramsthan	−20.214	57.737	798.95

**Fig. 4:**
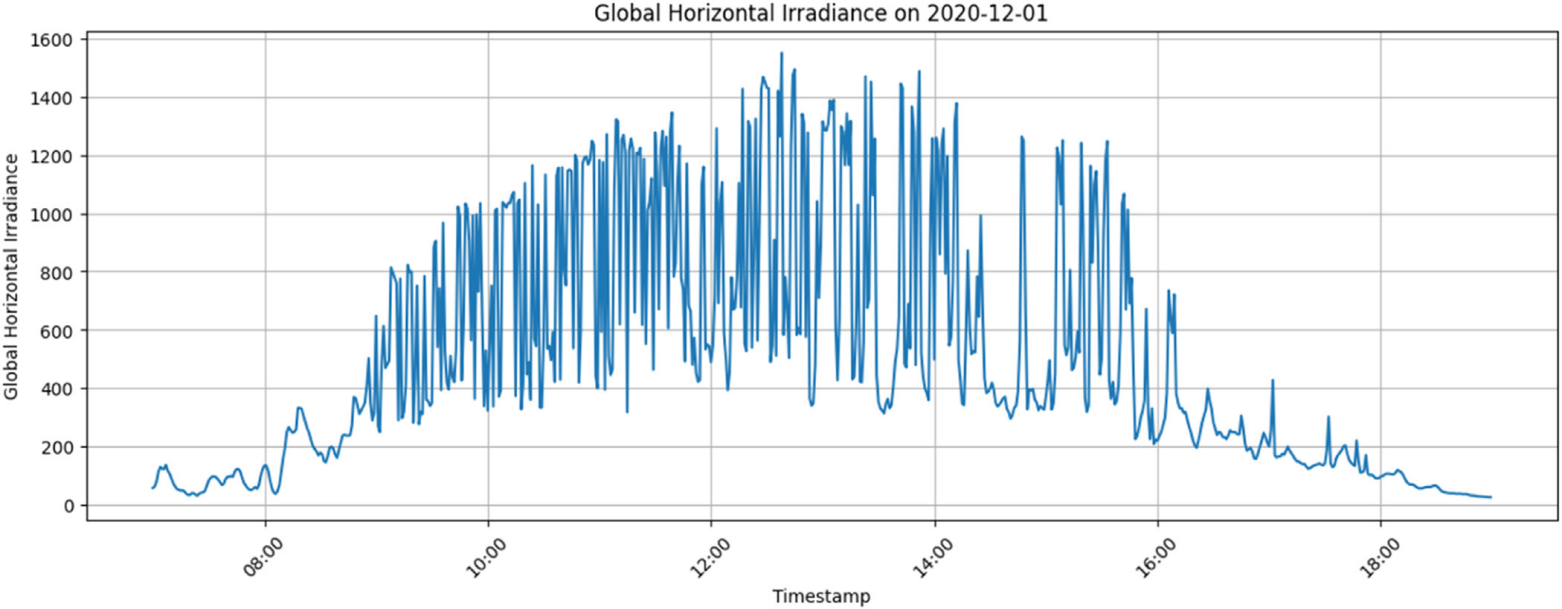
Global Horizontal Irradiance for Site Bramsthan on December 1, 2020.

**Fig. 5:**
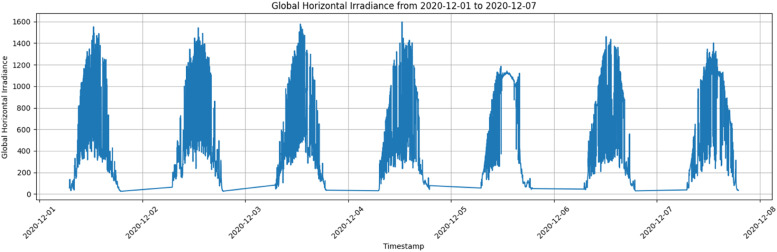
Weekly trend of Global Horizontal Irradiance for Site Bramsthan from December 1 to December 7, 2020.

**Fig. 6:**
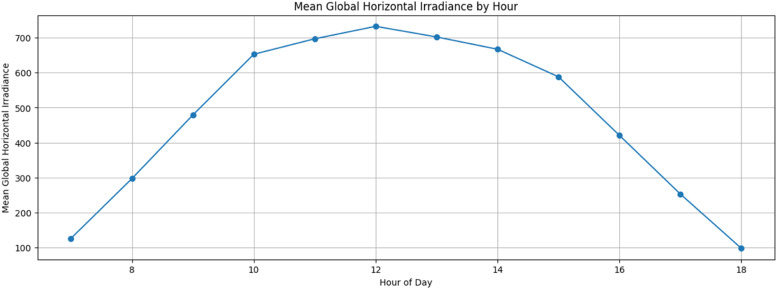
Average Daily Global Horizontal Irradiance by Hour at Site Bramsthan for December 2020.

Fig. 4, Fig. 5, Fig. 6 illustrate the data trends and provide visual representation for better understanding. The average Global Horizontal Irradiance by Hour for the Site Bramsthan for the month of December is depicted in Fig. 6.

The Global Horizontal Irradiance data for each site was organized into individual .csv files contained in the folder named by Site, with each Site stored within its respective island directory. These island directories, in turn, were stored within the main Data directory. Table 2 shows the structure of the main data directory.

**Table 2:** Directory structure of the main data directory.

Directory Tree	Description
∟ DATA/	directory of Global Horizontal Irradiance Data
⊢AGALEGA/	directory of Global Horizontal Irradiance Data for the island of Agalega
⊢MAURITIUS/	directory of Global Horizontal Irradiance Data for the island of Mauritius
∟RODRIGUES/	directory of Global Horizontal Irradiance Data for the island of Rodrigues

**Fig. 7:**
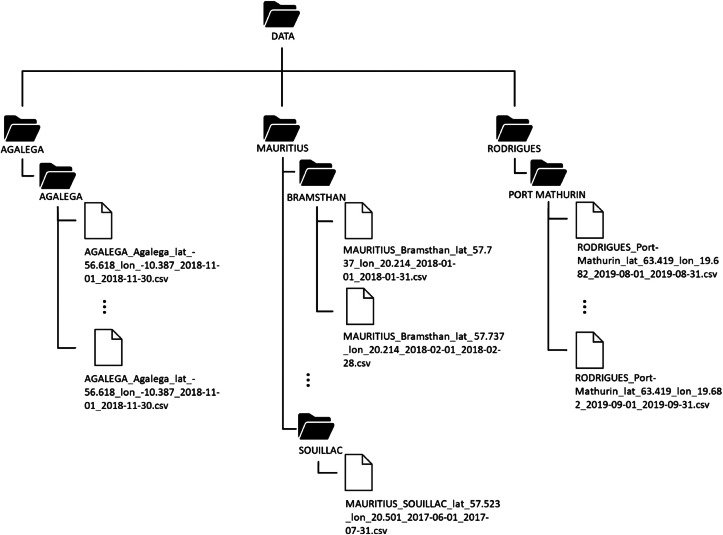
Data folder layout.

Fig. 7 illustrates the layout of the data directory to provide a better understanding of the file name format and the overall directory structure. The file name format for the csv files for each site is “{Island}_{Site}_lat_{latitude}_lon_{longitude}_ {date_from}_{date_to}.csv”. For example, the file “MAURITIUS_Bramsthan_lat_−20.214_lon_57.737_2018–01–01_2018–01–31”, represents data collected at Bramsthan, Mauritius at latitude “−20.214” and longitude of “57.737” from January 1,2018 to January 31,2018.

## 4 Experimental Design, Materials and Methods

**Fig. 8:**
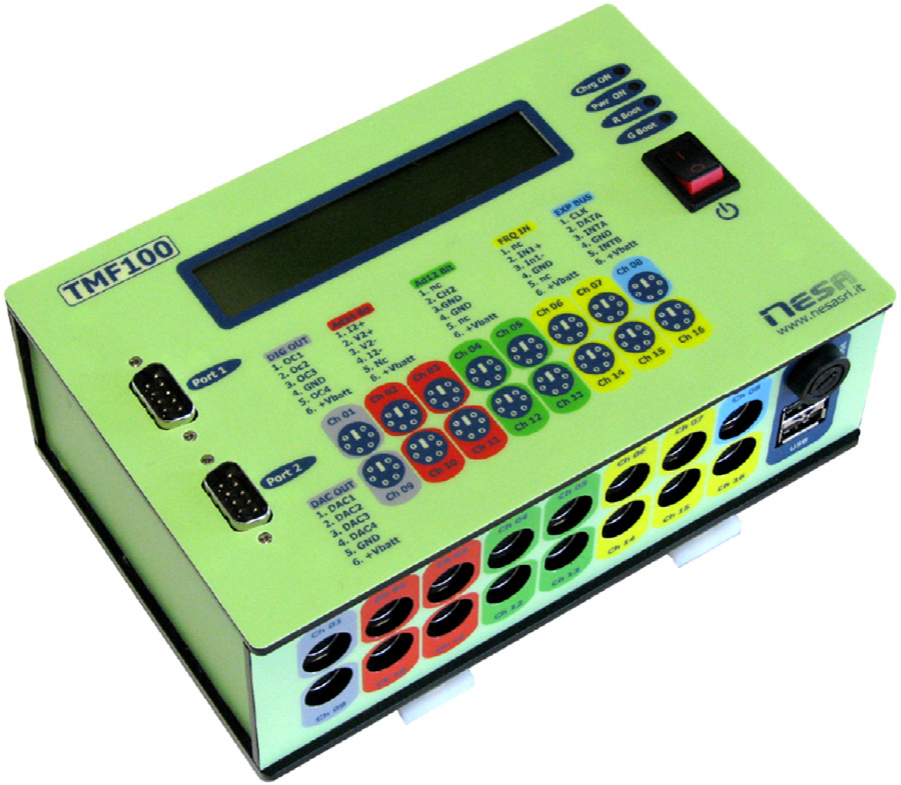
Photo of TMF100 Data Logger [2].
• Nesa M2CH sensor protection board [3].
• MS-802 EKO Pyranometer [4].
• Silicon Irradiance Sensor with Integrated temperature sensor [5].
• Air temperature sensor [6].

Base Stations were established across the twelve sites for the collection of Global Horizontal Irradiance (GHI) data. The Base Station responsible for data acquisition consisted of:

• TMF 100 Data Logger [2] (Fig. 8).

**Fig. 9:**
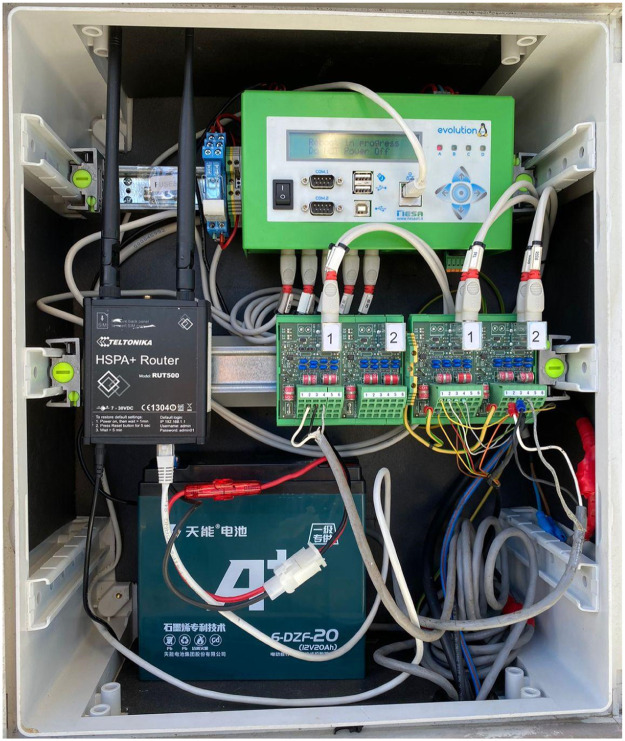
Photo of base station.

The TMF 100 Data Logger served as the central connection point for all sensors, including the pyranometer, Silicon Irradiance sensor, and Air temperature sensor. IP68 black connector cables with PS2 connectors were linked to the M2CH sensor protection board before being connected to the data logger to safeguard against overvoltage. Power was supplied through a system comprising an auxiliary power supply battery and a solar panel. Data transmission to an FTP server was done via a GPRS modem. Fig 9. Below shows the base station equipped with all of the sensors and the TMF100 data logger mounted.

The sensors were configured to record data at 1-hour intervals. Raw data was retrieved from the FTP server and cleaned to remove erroneous characters. The data, originally in .txt format and delimited with commas and hash, were processed as follows. The data was first read from the .txt file through the utilization of a Python script. Unnecessary data were filtered out, retaining only GHI values.

Following extraction, headers (Timestamps, Site Name, Latitude, Longitude, and Global Horizontal Irradiance) were added to the data. The data also underwent time alignment with an offset of eight hours and nighttime hours (before 7 and after 19) were removed. The resulting dataset was saved in .CSV format with filenames denoting the site's island, name, latitude, longitude, starting date, and ending date.

This procedure ensured the acquisition of accurate GHI data for analysis and interpretation.

## Limitations

When using the File Transfer Protocol, data transmission could be unreliable, resulting in missing or delayed data uploads.

FTP is an unencrypted protocol, making it vulnerable to security risks like data interception, unauthorized access, and tampering.

Transferring high-frequency solar irradiance data (especially from multiple sensors) could slow down the system, affecting data collection performance.

Data collection systems using FTP may face integration issues with cloud storage or real-time analysis tools, requiring additional effort for data synchronization.

## Ethics Statement

The current work does not involve human subjects, animal experiments, or any data collected from social media platforms.

## CRediT authorship contribution statement

**Yogesh Beeharry:** Conceptualization, Methodology, Project administration, Supervision, Funding acquisition, Writing – review & editing. **Ravish Gokool:** Data curation, Writing – original draft. **Yatindra Kumar Ramgolam:** Conceptualization, Methodology, Project administration, Funding acquisition, Writing – review & editing. **Aatish Chiniah:** Methodology, Project administration, Writing – review & editing.

## Data Availability

Global Horizontal Irradiance Dataset for Mauritius, Rodrigues, and Agalega Islands (Original data) (Science Data Bank)
